# Extracellular Vesicles As Mediators of Cardiovascular Calcification

**DOI:** 10.3389/fcvm.2017.00078

**Published:** 2017-12-11

**Authors:** Amirala Bakhshian Nik, Joshua D. Hutcheson, Elena Aikawa

**Affiliations:** ^1^Department of Biomedical Engineering, Florida International University, Miami, FL, United States; ^2^Center for Interdisciplinary Cardiovascular Sciences, Brigham and Women’s Hospital, Boston, MA, United States; ^3^Cardiovascular Division, Department of Medicine, Center for Excellence in Vascular Biology, Brigham and Women’s Hospital, Harvard Medical School, Boston, MA, United States

**Keywords:** calcification, atherosclerosis, aortic stenosis, hyperphosphatemia, extracellular vesicles, matrix vesicles

## Abstract

Involvement of cell-derived extracellular particles, coined as matrix vesicles (MVs), in biological bone formation was introduced by Bonucci and Anderson in mid-1960s. In 1983, Anderson et al. observed similar structures in atherosclerotic lesion calcification using electron microscopy. Recent studies employing new technologies and high- resolution microscopy have shown that although they exhibit characteristics similar to MVs, calcifying extracellular vesicles (EVs) in cardiovascular tissues are phenotypically distinct from their bone counterparts. EVs released from cells within cardiovascular tissues may contain either inhibitors of calcification in normal physiological conditions or promoters in pathological environments. Pathological conditions characterized by mineral imbalance (e.g., atherosclerosis, chronic kidney disease, diabetes) can cause smooth muscle cells, valvular interstitial cells, and macrophages to release calcifying EVs, which contain specific mineralization-promoting cargo. These EVs can arise from either direct budding of the cell plasma membrane or through the release of exosomes from multivesicular bodies. In contrast, MVs are germinated from specific sites on osteoblast, chondrocyte, or odontoblast membranes. Much like MVs, calcifying EVs in the fibrillar collagen extracellular matrix of cardiovascular tissues serve as calcification foci, but the mineral that forms appears different between the tissues. This review highlights recent studies on mechanisms of calcifying EV formation, release, and mineralization in cardiovascular calcification. Furthermore, we address the MV–EV relationship, and offer insight into therapeutic implications to consider for potential targets for each type of mineralization.

## Background

Bonucci reported the appearance of “roundish bodies” in initiation of the calcification process in guinea pig and rat tibial–femoral epiphyseal plates in 1967 (1). One year later, Anderson used electron microscopy on tissue sections to demonstrate vesicular structures in the mouse cartilage epiphysis (2). Subsequent studies have suggested that mineralization depends on secretion of *matrix vesicles (MVs)*, with diameter of 30–400 nm (3, 4), from chondrocytes, osteoblasts, odontoblasts, tenocytes, and cementoblasts (5). MVs released from specific sites on cell membranes [apical microvilli (3)] exhibit similar cytosolic and plasma membrane profiles apparent in their cell of origin (6). Compared to their parent cells, MVs carry augmented levels of acidic lipids such as phosphatidylserine (PS) and sphingomyelin, but diminished levels of neutral phospholipids of phosphatidylcholine and lysophospholipids (5). Chondrocytes residing in the epiphyseal plate experience hypoxic conditions due to presence of collagen fibrils and proteoglycans, which restrict oxygen and nutrients delivery (7). Development of blood microvessels into this zone creates oxidative stress caused by the sudden elevation in nutrients, oxygen, calcium ions (Ca^2+^), and phosphate ions (Pi) (8). This process leads to enrichment of mitochondria with Ca^2+^, which results in secretion of Ca^2+^-loaded vesicles into the cytosol. It has been proposed that the formation of complexes between PS and Ca^2+^ either with Pi or annexins in these vesicles, diminishes the Ca^2+^ level within the cytosol, expedites actin depolymerization, and consequently leads MVs to pinch-off from the cell and release into the extracellular environment (8, 9). Of note, apoptotic bodies originating from apoptotic cell membrane rearrangement during terminal stage of mineralization in epiphyseal plate differs from active formation and release of MVs (10). Released MVs interact with glycosaminoglycans and initiate extracellular mineral deposition (11).

Chondrocyte differentiating factors such as thyroxine (T3), bone morphogenetic protein 6, retinoic acid, and Indian hedgehog may give rise to MV generation by inducing changes in cell phenotype (12). Osteogenic cell types abundantly express annexin I, II, IV, V, VI, and VII. Annexins function as voltage-gated channels or Ca^2+^-binding agents, mediate inflammation responses, and regulate structural properties of both cells and MVs membranes (4, 11, 13). The most abundant proteins in MVs are annexins II, V, and VI, which can accelerate the calcification process by either providing required Ca^2+^ for mineralization or partaking in PS–Ca^2+^-annexin complexes (14). Additionally, other membrane proteins such as calbindin D9k can provide Ca^2+^ for MVs (15).

Pyrophosphate (PPi), which originates from nucleotide pyrophosphatase phosphodiesterase (NPP1) hydrolysis of nucleoside triphosphates, inhibits mineralization. Progressive ankylosis (ANK) carries PPi into extracellular milieu. Tissue non-specific alkaline phosphatase (TNAP, on the outer leaflet of MV membranes) hydrolyzes PPi into free phosphate (Pi) and provides free Pi for complexing with Ca^2+^ and mineral formation (5, 7, 16). Type III Na/Pi transporters (PiT-1) on the MV membrane facilitate Pi internalization. Bone morphogenetic protein 2 and parathyroid hormone upregulate expression of these transporters (7). Additionally, MVs contain phosphatases and membrane phosphohydrolases, such as TNAP, AMPase, ATPase, nucleoside triphosphate pyrophosphohydrolase (NTPPase, NPP1, and PC-1), and PHOSPHO-1 that elevate the intravesicular Pi concentration within the MVs (5, 17). This highly ions concentrated environment, adjacent to the MV membrane, where Ca^2+^ and Pi meet, provokes calcium phosphate precipitation followed by an increase in pH and mineral stabilization. Membrane–mineral associations are mediated by PS located on the luminal side of the MV lipid bilayer. PS is an anionic phospholipid with tendency to bind Ca^2+^. The complexes of PS–Ca^2+^–Pi may serve as initial nuclei for hydroxyapatite precipitation (7, 13, 18). Studies have also suggested that the mineralization process can begin intracellularly with contribution of pre-nucleation clusters within endosomes, which initiate mineralization in order to maintain Ca^2+^ and Pi in homeostatic concentration and fulfill energy trade-off (5).

**Figure 1:**
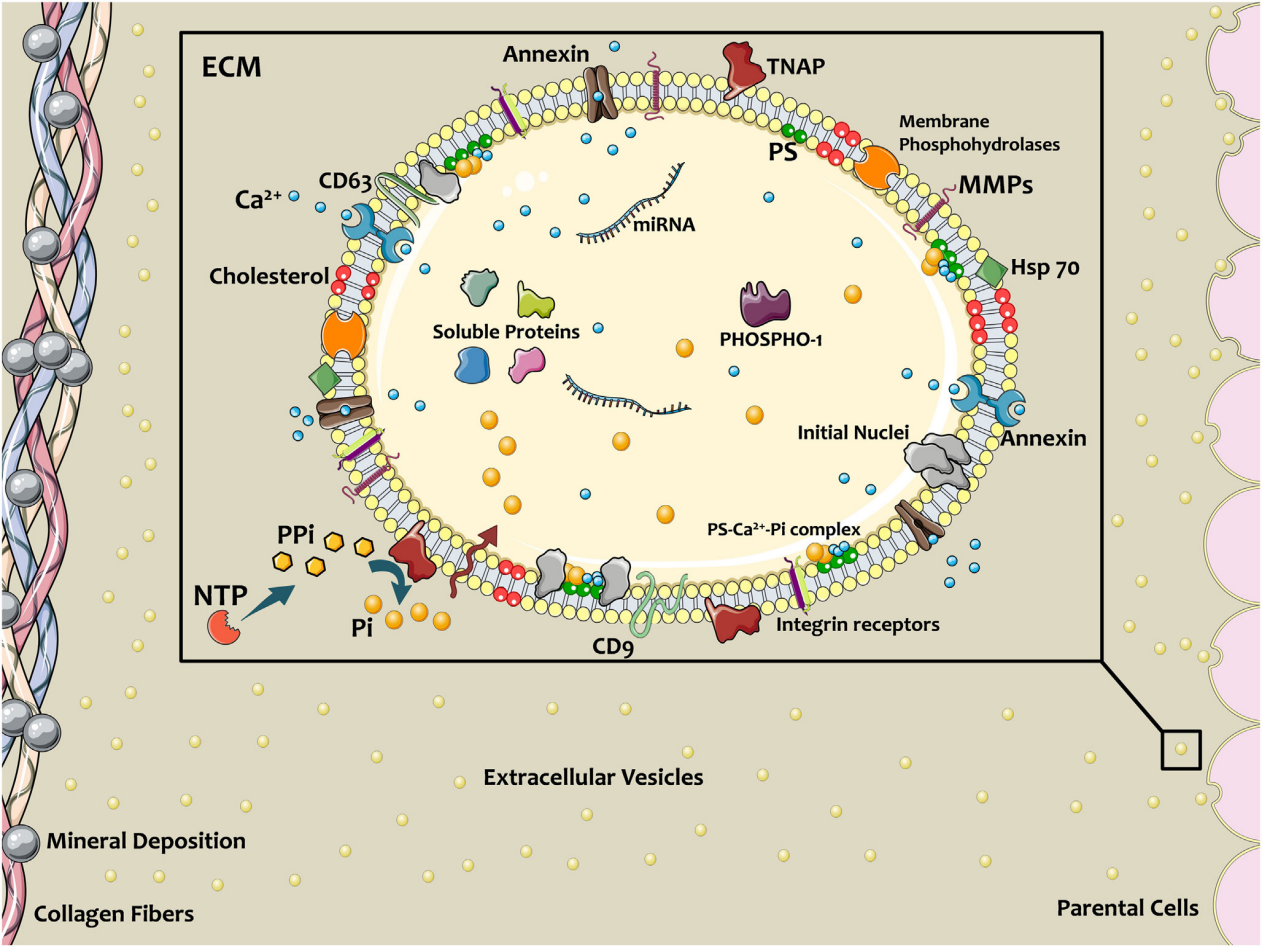
Schematic of extracellular vesicles (EVs) contributing in calcific mineral formation. Osteogenic cells release EVs into ECM to nucleate bone or cardiovascular mineral. EV membranes contain a specific lipid profile that differs from the parent cell. Annexins on EVs facilitate Ca^2+^ entrance, and tissue non-specific alkaline phosphatase (TNAP) activity converts PPi to phosphate ions (Pi), thereby providing the necessary ionic components for mineralization. PiT-1 transporters transfer Pi into the EV lumen. Coincidence of these ions and formation Ca^2+^–PS–Pi within the EV lead to mineral initiation. Membrane enzymes and proteins interact with and attach to the ECM, directing the localization of calcification. Figure created using Servier Medical Art images (http://smart.servier.com).

Matrix vesicles are equipped with matrix metalloproteases (MMPs) that degrade and remodel the ECM (3, 19). Particularly, MMP-2, MMP-3, MMP-9, and MMP-13 are located in MV membranes and play a key role in matrix remodeling and propagation of mineralization (5, 19, 20). In ECM, collagen fibrils and proteoglycans provide charged regions, which are favorable sites for accumulation of the calcium phosphate nanoparticle clusters that form in MVs. The amorphous apatite resulting from this accumulation transforms into structured and crystalline mineral following ECM associations (5). MVs interact with ECM proteins *via* their integrin receptors and surface motifs, such as CD9, CD63, and Hsp70 (5) (Figure 1). In addition to their remodeling potential and ECM binding, MVs in growth plate ECM can affect the proliferation and fate of resident cells, due to the activation of parathyroid hormones and gene-related peptide through their loaded proteins (5). This paracrine signaling property is similar to other subtypes of extracellular vesicles (EVs) ubiquitous to many cells and tissues.

It is worth mentioning that skepticism exists on the existence and role of MVs in bone growth and formation. Studies have suggested that calcification originates from cell death and debris, which nucleate mineral, and observations of MVs are attributed to sample preparation artifacts (21). In recent studies, however, isolation of MVs from chicken tibias using multiple centrifugations and application of microscopy techniques such as transmission electron microscopy on growth plate during bone formation have provided support for MVs as mediators of mineralization (22, 23). Furthermore, knockout of Stx4a, a regulatory factor for secretion of MVs, decreases number of MVs and leads to reduced bone mineral density (24). Recent interest in non-calcifying EVs (e.g., exosomes) and the related development of assay techniques may build on the observational studies of the past and help clarify the derivation and function of MVs in the mineralization of hard tissues.

## EVs with Non-Osteogenic Origins

Matrix vesicles represent one specific subtype of EVs. Generally, EVs function to maintain both intracellular and extracellular homeostasis. Two major pathways mediate EV release into the ECM: (i) *via* multivesicular bodies (MVB), containing several EVs wrapped by plasma membrane and (ii) through direct budding of a single EV from cellular plasma membrane (4). Depending on the release mechanism, EVs are generally referred to as exosomes and microparticles, respectively. Both EV types carry a subset of cargos representing their parental cell (11, 25). MVB trafficking and fusion/fission from the plasma membrane requires activity of Rab GTPases, specifically Rab27a, Rab27b, Rab35, and Rab11 (26, 27). EV structure consists of a metabolically active membrane with transmembrane proteins and an inner core, which typically carries RNAs, soluble proteins, and lipids (11, 25, 28). EVs often contain sets of small RNAs, such as miRNA, tRNA, mRNA, piRNA, snRNA, Y-RNA, and vault RNA, which can be protected from RNase degradation and encoded at target cells (27, 29). The difference in RNA ingredients of parental cells and EV cargo demonstrates the selective mechanism of RNA loading. EV RNA cargo can serve as biomarker that indicate the phenotypic state of parental cells, as well as messengers that can interact with other cells (27). EVs play a key role in cell–cell interaction and data trafficking in both normal and pathological conditions. For instance, miRNA-enriched EVs from endothelial cells can regulate gene expression and resultant phenotypic transitions in smooth muscle cells (SMCs) (30). Divergence from normal physiological conditions toward pathological ones induces release of dysfunctional EVs with pathologic cargo and may affect tissue homeostasis and cellular phenotypes (4). This section serves as a short primer on the complex mechanisms associated with EV release and cargo loading. These processes are reviewed in greater detail elsewhere (31–34). The current review focuses on EVs that play a direct role in depositing mineral in cardiovascular tissues. Current evidence, discussed in the following sections, suggests that these EVs share commonalities with both MVs and other EV populations, such as exosomes.

## Calcifying EVs in Cardiovascular Calcification

Calcification contributes to pathological remodeling in different locations throughout the cardiovascular system, such as the arterial intima and media and the aortic valve (35, 36). Electron microscopy demonstrated the presence of needle-like hydroxyapatite nanocrystals in EVs extracted from atherosclerotic lesions of apolipoprotein E-deficient mice (37). EVs released into the atherosclerotic lesion have a Ca/P ratio of 0.66, indicating incomplete calcification [compared to the ratio in normal adult murine bones of 1.71 (38)]. High resolution microcomputed tomography imaging revealed microcalcification in the fibrous cap of atherosclerotic plaques composed of calcified EV aggregates (39, 40). These observations indicate a role for EVs in the formation and progression of cardiovascular calcification, but mechanistic studies demonstrating causality are difficult. One major challenge in EV research is to distinguish between different EV populations, such as calcifying EVs, MVs, exosomes, apoptotic bodies, and microparticles, due to their shared size and shape characteristics (41). Application of multiple and consecutive centrifugations followed by size-based filtration and sucrose gradient-based ultracentrifugation to isolate EVs of known density have been used to separate different cellular-derived particles and EVs (25, 42). EVs with various sizes and densities pellet based on the centrifugation speed, i.e., large, medium, and small EVs precipitate at low, moderate, and high speeds, respectively (25). However, these techniques are often unable to separate the various EV populations. Calcifying EVs secreted by SMCs cultured under osteogenic conditions exhibit increased mass density compared to other EVs, likely due to mineral formation. Therefore, calcifying EVs precipitate more quickly under the application of ultracentrifugation (~100,000*g*) (41), permitting enrichment of these EVs and subsequent proteomic profiling (25, 41). Once proteomic fingerprints are established, membrane proteins (tetraspanins), ER-related proteins, mitochondrial proteins, exosomal markers, endosomal markers, and ECM factors (such as surface molecules and integrins) specific to various EV populations help map and separate EVs into subsets based on their origins (25).

In certain pathological conditions (such as chronic kidney disease), extracellular Ca^2+^/Pi concentrations increase, thereby cells release EVs with high levels of these ions (43). This may be in contrast to inflammation-driven osteogenic differentiation (e.g., in atherosclerosis). Release of EVs from cardiovascular cells with osteogenic phenotypes promotes mineralization *via* generation of free phosphate from sources such as ATP and pyrophosphate (44). Further, dystrophic mineral deposition resulting from cell death may also contribute to a significant portion of cardiovascular calcification (45). The diverse contributors have led to confusion surrounding the mechanisms of cardiovascular calcification (46); however, numerous studies have demonstrated similarities between cardiovascular calcification and bone metabolism (47–51). Annexins present on calcifying EVs play a dual role of Ca^2+^ uptake and counteracting the calcification inhibitory activity of fetuin-A during ectopic mineralization (4, 11). High phosphate imbalance present in chronic kidney disease may accelerate calcification nucleation in EVs (46). In this condition, TNAP activity may accelerate the calcification process in both non-osteogenic and osteogenic EVs through removing PPi, a competitive inhibitor to Ca^2+^ associations with Pi (39). EVs with calcifying potential may also contain imbalanced and dysfunctional miRNAs, which induce the gene expression and protein synthesis of osteogenic markers, such as RUNX2, Smad1, osterix, TNAP, chaperones, and pro-inflammatory factors (4, 11). Unlike the physiological process of MV-mediated mineral deposition, however, calcification of EVs may inhibit their ability to reach their intended target cells, further promoting a loss in tissue homeostasis and pathological remodeling (52) (Figure 1).

Future works are needed to improve upon EV isolation techniques in order to reduce noise from non-calcifying EVs. Advancements in EV characterization tools may allow careful comparative studies to understand the similarities and differences between MVs and calcifying EVs liberated from the three reported cellular contributors to calcification in cardiovascular tissues: vascular SMCs, macrophages, and valvular interstitial cells (VICs). While these cell populations do not represent all cells involved in cardiovascular calcification, direct EV-related contributions to mineral nucleation from other cells remain unreported. Improvement of EV isolation and assay techniques may also allow for determination of the relative roles of the various cell populations in the mineralization process.

### Smooth Muscle Cell-Derived Vesicles

Smooth muscle cells and osteoblasts share similar mesenchymal origins and under pathological stresses SMCs can exhibit an osteoblast-like phenotype (48). In a hyperphosphatemic environment (e.g., chronic kidney disease) or inflammation-driven atherosclerosis, vascular SMCs upregulate expression of osteogenic differentiation genes (53) and release EVs enriched with pro-calcific biomarkers (44). Hyperphosphatemic and osteogenic conditions decrease circulating factors, such as fetuin-A (51) and matrix Gla protein (50), which inhibit extracellular mineralization, but increase TNAP and annexins II and VI in SMC-derived EVs, which promote extracellular mineralization. Similar to its function in bone, TNAP activity leads to increased available Pi and reduction of mineralization inhibitors such as PPi (49). The formation of calcifying EVs begins with a series of intracellular trafficking processes that produce the EVs with calcification-promoting factors. A specific trafficking protein, sortilin, is a key player in the formation of calcifying EVs secreted by vascular SMCs. Sortilin transports TNAP into SMC-derived EVs, thereby increasing EVs calcifying potential (49).

**Figure 2:**
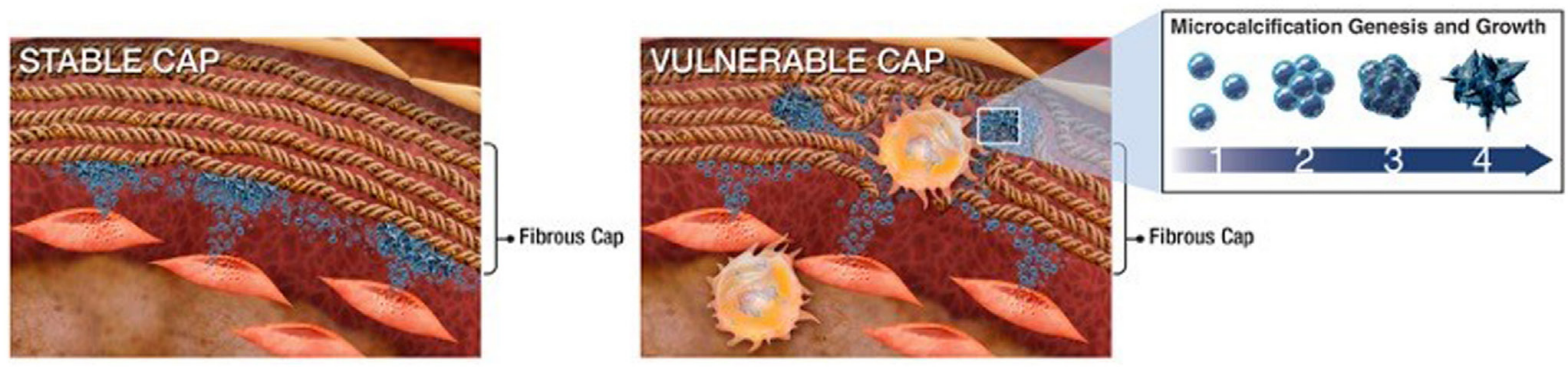
Schematic of extracellular vesicle (EV)–collagen interaction to form microcalcifications in the atherosclerotic fibrous cap. Collagen degradation in vulnerable fibrous caps allows calcifying EVs to accumulate. The initial mineral nuclei forms within individual EVs and EV aggregation and fusion drives mineral maturation and growth (36).

Materials science imaging techniques revealed that, once released into the ECM, SMC-derived calcifying EVs tend to aggregate and form microcalcifications in areas with sparse collagen, whereas, large calcifications (larger than 200 µm) are bordered by dense collagen fibers (36). Large calcifications (macrocalcifications), shaped by accumulation of small calcifications (microcalcifications), gradually form mature mineral. Microcalcifications formed within the fibrous cap of vulnerable atherosclerotic plaques further potentiate plaque rupture, whereas larger macrocalcifications beneath stable fibrous caps may promote plaque stability. EVs collected from SMCs cultured in pro-calcific conditions and incubated within an *in vitro* collagen hydrogel system, mimicking aspects of atherosclerotic lesions, showed the progression of calcification from single calcifying EVs to EVs aggregation and fusion to the formation of microcalcifications to growth into large calcifications (36). Collagen acts as a scaffold to direct the shape and size of the calcifications generated from this growth process (36). Collagen fibrils, specifically type I and III in arteries, bind to EV membranes and mediate calcification propagation in the ECM (48). Figure 2 illustrates the mechanism by which EVs associate to form mineral within the tissue, beginning with EV accumulation and aggregation to fusion and mineralization. In addition to binding and directing the calcification of EVs, collagen may also play a role in phenotypic changes and EV formation within SMCs (54). Discoidin domain receptor-1 (DDR-1), a collagen receptor, regulates SMC phenotype by sensing extracellular collagen. DDR-1-depleted SMCs exhibit elevated collagen production, EV release, and mineral deposition. DDR-1 functions as a regulator of TGF-β signaling pathways, acting as a switch between pro-fibrocalcific and anti-fibrocalcific TGF-β signaling (54). Therapeutic strategies to control these pathways and prevent or reverse SMC-driven calcification will require a better understanding of the mechanisms that lead to their formation within the cell, nucleation of mineral outside the cell, and the role of the ECM in calcification propagation. SMC-derived EVs are the most studied type of calcifying EVs in cardiovascular tissues and the mechanisms identified in these cells may help inform research into other cellular drivers of cardiovascular calcification.

### VIC-Derived Vesicles

Valvular interstitial cell phenotypic changes play a vital role in ECM remodeling and mineral deposition that lead to calcific aortic valve disease (55). VICs have a high phenotypic plasticity and can transform from a fibroblastic phenotype to myofibroblast- or osteoblast-like cells in response to pathological conditions, such as hyperphosphatemia and pro-inflammatory cytokines. VICs exhibit high sensitivity to their mechanical environment and undergo phenotypic changes *in vitro* in response to changes in substrate stiffness and mechanical stretch (55). VICs may also influence valvular endothelial cells (VECs) through EV secretion. Valve homeostasis depends on appropriate interactions between VECs and VICs (55, 56). This intercellular interaction occurs when VECs take up VICs-derived EVs containing perinuclear proteins (35). In calcifying milieu, VICs express osteogenic mRNAs of PiT-1, RUNX2, Msx2, and TNAP (57), and pro-calcific VIC-derived EVs resemble MVs from chondrocytes and SMCs, demonstrating elevated annexins II, V, and VI (35). Similarly, immortalized rat VICs cultured in high calcium and phosphate media release EVs with elevated calcium and annexin VI, and electron microscopy revealed co-localization of annexin VI with EVs in calcified human aortic valves (57). Though aortic valve calcification constitutes a major unmet clinical problem, investigations into the extracellular mechanisms through which mineral nucleation and growth occurs remain scant. More studies are needed to understand the role of VIC EVs in this process and the similarities and differences between these EVs and the more well-studied SMC-derived EVs.

### Macrophage-Derived Vesicles

**Figure 3:**
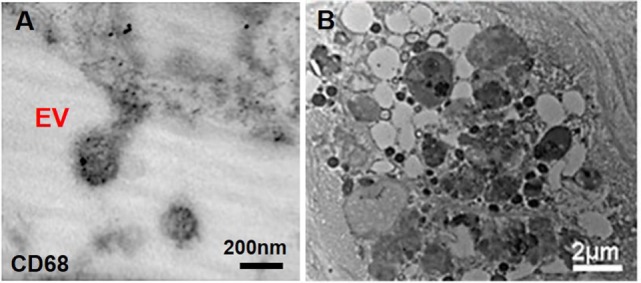
Macrophage-derived extracellular vesicles (EVs) within atherosclerotic cap. **(A)** Macrophage-derived EVs contain the CD68 glycoprotein (immunogold staining); **(B)** released EVs have various size and morphology and aggregate to build calcific mineral within the atherosclerotic fibrous cap (63).

Atherosclerosis creates moderate hypoxia (2% oxygen) for local cells and leads to activation of pro-inflammatory responses such as Akt and β-catenin pathways in macrophages (58). Additionally, oxidized lipids, IL-6, and TNF-α (pro-inflammatory cytokines), and mechanical stimuli contribute to both increased inflammation and subsequent ectopic calcification (59). Pro-inflammatory macrophages secrete elastolytic cathepsins and collagen-degrading MMPs (e.g., MMP-2 and -9), and the resultant proteolytic ECM degradation and remodeling causes atherosclerotic plaque instability and rupture, the leading cause of cardiovascular morbidity (60, 61). Inflammation precedes and may serve as a requisite step for the onset of both atherosclerotic and aortic valve calcification (61). Monocytes internalize forming minerals and secrete more inflammatory cytokines and intensify pathologic condition (62). Cytokines secreted by pro-inflammatory macrophages exacerbate calcification by activating apoptosis or osteogenic pathways activation in SMCs and VICs (46). In addition to an indirect role in pro-calcific remodeling, macrophages can directly contribute to cardiovascular calcification through release of calcifying EVs in hyperphosphatemic milieu (46). Macrophage-derived calcifying EVs contain the tetraspanin exosomal markers of CD9, CD63, CD81, and TSG101 (28, 63). These EVs also exhibit immuno-positivity for CD68 (63) (Figure 3A). In EVs released by macrophages, calcium mineral nucleates on complexes containing S100A9 [a pro-inflammatory and pro-thrombotic factor (11)], PS, and annexin V on the EV membrane (4). Accumulation and aggregation of these EVs results in mineral growth within atherosclerotic plaques (Figure 3B). Of note, macrophages contribute to both vascular and valvular calcification; therefore, macrophage-derived EV calcification could provide a link between mineral depositions within these tissues.

## Conclusion

Calcifying EVs play an important role in the initiation and development of cardiovascular calcification. Though calcifying EVs in cardiovascular tissues appear to share commonalities with MVs, they seem to be derived from different origins within cells. The overlapping characteristics between EVs and MVs underscore the fact that research in cardiovascular calcification has been informed by pioneering research in bone physiology. However, the noted differences between cardiovascular calcifying EVs and bone MVs warrant further investigation. The type and quality of mineral that forms appear different in the two tissues. Further, the appearance of calcific mineral in cardiovascular tissues associates strongly with a decrease in bone mineral density—a phenomenon known as the calcification paradox (59). Studies into the differences between the fundamental building blocks of calcification—calcifying EVs in cardiovascular tissues and MVs in bone—may provide new insight into the observed divergence in mineral within these tissues and present therapeutic options that avoid unwanted off-target effects.

## Author Contributions

AN researched the topic and drafted the manuscript. JH helped draft the original manuscript and edited and revised the manuscript text. EA provided intellectual contributions to the original manuscript draft and substantially edited and revised the manuscript text.

## Conflict of Interest Statement

The authors declare that the research was conducted in the absence of any commercial or financial relationships that could be construed as a potential conflict of interest.
